# Contextual factors associated with diarrhea among under-five children in the Gambia: a multi-level analysis of population-based data

**DOI:** 10.1186/s12879-024-09350-9

**Published:** 2024-05-09

**Authors:** Amadou Barrow, Solomon P.S. Jatta, Oluwarotimi Samuel Oladele, Osaretin Godspower Okungbowa, Michael Ekholuenetale

**Affiliations:** 1 Department of Public & Environmental Health, School of Medicine & Allied Health Sciences, University of The Gambia, Kanifing, The Gambia; 2 Department of Epidemiology, College of Public Health & Health Professions, University of Florida, Gainesville, FL USA; 3 Amrita School of Sustainable Development, Amrita Vishwa Vidyapeetha University, Kollam, Kerela India; 4 School of Public Health, Gambia College, Brikama, The Gambia; 5 Department of Demography and Social Statistics, Faculty of Social Sciences, Federal University Oye-Ekiti, Oye-Ekiti, Ekiti State Nigeria; 6 Department of Economic and Social Research, National Institute for Legislative and Democratic Studies, National Assembly, Abuja, Nigeria; 7 Department of Economics, Faculty of Social Sciences, University of Benin, Benin City, Nigeria; 8 Department of Epidemiology and Medical Statistics, Faculty of Public Health, College of Medicine, University of Ibadan, Ibadan, Nigeria

**Keywords:** Diarrhea, Contextual factors, Gambia, Multi-level, Under-five children

## Abstract

**Background:**

Diarrhea poses a significant threat to the lives of children in The Gambia, accounting for approximately 9% of all deaths among children under the age of five. Addressing and reducing child mortality from diarrhea diseases is crucial for achieving the Sustainable Development Goal (SDG) 3, specifically target 3.2, which aims to eliminate preventable deaths in newborns and children under the age of five by 2030. Thus, this research aims to assess the prevalence and contextual factors associated with diarrhea among under-five children in The Gambia.

**Methods:**

This research employed secondary data from the 2019/20 Gambia Demographic Health Survey (GDHS). The study initially involved 8,362 women aged between 15 and 49 years. Of these, 6,929 women with children under five were included in this analysis. Data were analyzed using STATA with cross-tabulation and model fitting. Multilevel logistic regression was applied to accommodate the hierarchical structure of the demographic health survey data. The model comparison parameters were BIC, AIC, deviance, and LLR. Variables with a *p*-value less than 0.05 were selected for multivariable analysis. The statistical significance of the factors was determined using an adjusted odds ratio with a 95% confidence interval (CI) and a *p*-value of less than 0.05.

**Results:**

The prevalence of diarrhea in under-five children was 53.2% in males and 46.8% in females. In the final model, Kerewan (aOR = 0.58; 95% CI = 0.33–0.98) and Basse (aOR = 0.59; 95% CI = 0.35–0.98) have significantly lower odds of childhood diarrhea compared to Banjul, female children show slightly lower, yet significant, odds of diarrhea compared to males (aOR = 0.96; 95% CI = 0.86–0.98), deliveries at government health centers are associated with higher odds of childhood diarrhea compared to home births (aOR = 1.24; 95% CI = 1.01–1.52). Mothers with post-secondary education had significantly lower odds of having children with diarrhea than those without any education (aOR = 0.50; 95% CI = 0.26–0.99) after controlling for confounders.

**Conclusion:**

The study findings indicate that several factors significantly impact the risk of childhood diarrhea in The Gambia. These factors include region of residence, sex of the child, place of delivery, and education level of the mother. The study suggests that existing interventions aimed at improving child health outcomes in the country should take into consideration these influential factors. Addressing these modifiable factors can enhance the effectiveness of interventions and promote better health outcomes for children in Gambia.

**Supplementary Information:**

The online version contains supplementary material available at 10.1186/s12879-024-09350-9.

## Background

Diarrhea is the passage of three or more watery or loose stools within 24 hours [1]. It is the second most common cause of death in children under the age of five years, behind pneumonia, and it kills at least one in nine children or in excess of 525 000 children each year [1]. It is estimated that by 2030, 4.4 million children under the age of five years would die each year from complications associated with infectious illnesses, with Sub-Saharan Africa accounting for 60% of these deaths [2]. Diarrhea accounts for approximately 3.6% of the global disease burden, as measured by disability-adjusted life years (DALYs) [3]. Despite a significant reduction in global mortality caused by diarrhea in the past 25 years, its morbidity has not experienced a similar decline. This can be attributed to persistently high levels of risk factors, including inadequate access to clean water, sanitation, and hygiene (WASH), insufficient promotion of breastfeeding, and prevalent malnutrition. These challenges are particularly pronounced in low- and middle-income countries [4]. In order to mitigate the occurrence of diarrhea, the World Health Organization (WHO) has emphasized the importance of ensuring access to safe drinking water, adequate sanitation facilities, and practice of hand hygiene through the use of soap [5].

The integrated Global Action Plan for Pneumonia and Diarrhea advocated a holistic approach to ending unnecessary deaths from pneumonia and diarrhea, including WASH interventions [6, 7]. As a result of these efforts, the WHO/UNICEF Joint Monitoring Program (JMP) was established to evaluate the status of WASH in families, schools, and healthcare institutions on a global scale [8]. Most deaths from diarrhea are caused by severe dehydration and fluid loss. Children who are malnourished or have a weakened immune system are at the greatest risk of developing life-threatening diarrhea [9, 10]. Diarrheal diseases account for (82%) more than four-fifths of all deaths in children under the age of five in Africa and the Southern countries of Asia [11]. Approximately half of all deaths caused by pneumonia and diarrhea occur in just five of the world’s poorest countries: India, Nigeria, Democratic Republic of Congo, Pakistan, and Ethiopia [12, 13]. Reducing child mortality from diarrhea diseases is critical for achieving the Sustainable Development Goal (SDG) 3 (target 3.2), which aims to eliminate preventable deaths in newborns and children under the age of five by 2030 [14]. As a result, data on illness predictors and household health-seeking behaviors are critical for developing strategies targeted at reducing disease burden.

Diarrhea is a significant contributor to mortality among children in The Gambia, constituting approximately 9% of total deaths in children below the age of five [15]. Rotavirus is identified as the predominant etiological agent responsible for severe and fatal cases of diarrhea among young children globally. In The Gambia, this viral infection is responsible for a significant number of deaths and hospitalizations, specifically affecting approximately one-third of all cases of diarrhea disease among children under the age of five. It has been estimated that nearly 300 children in this age group succumb to rotavirus-related complications annually in The Gambia [15]. The Global Enteric Multicenter Study (GEMS) has confirmed the public health significance of rotavirus in The Gambia as it is identified as the primary cause of moderate-to-severe diarrhea in children below the age of two [16] and has revealed a significant association between Cryptosporidium infection and a heightened occurrence of moderate to severe infectious diarrhea among children within the age range of 0 to 24 months. According to the GEMS study [16], Cryptosporidium was identified as a significant cause of gastrointestinal illness in eastern Gambia, responsible for 12% and 8% of cases in children aged 0–11 months and 12–23 months, respectively. The GEMS study was a comprehensive investigation conducted over several years, employing standardized diagnostic protocols to assess the prevalence, distribution, and determinants of various infectious agents associated with diarrhea diseases [17–19].

In another study that aimed to assess the understanding of rural Gambians on diarrhea disease, it was observed that there was a high prevalence of diarrhea in children under the age of five in a defined group under demographic surveillance. It was concluded that inadequate healthcare facilities and practitioners as well as a lack of awareness in recognizing early indicators of dehydration are major issues amenable to public health interventions [20]. Thus, this research aims to assess the prevalence and contextual factors associated with diarrhea among under-five children: a multi-level analysis of population-based data in The Gambia.

## Methods

### Data source and study design

The present study employed a cross-sectional analysis methods to examine population-based data derived from the 2020 Gambia Demographic and Health Survey (GDHS). GDHS is a comprehensive survey conducted at the national level. It aims to gather data on various sociodemographic and health-related indicators, including but not limited to childhood diarrhea. Data was collected from the entire administration of Gambia using a sampling procedure consisting of three stages. The primary sampling unit of the survey comprised randomly selected samples from clusters. The study conducted in 2019 involved the participation of 8,362 women between the ages of 15 and 49. This study included a total of 6,929 women who had children under the age of five. The data was obtained from mothers or caregivers of infants who were born alive within the five years prior to the interview date.

### Study variables

#### Dependent variables

The dependent variable in this study was the occurrence of childhood diarrhea.

### Explanatory variable

The possible variables associated with childhood diarrhea were categorized into three levels: child level, mother level, and household level. These variables were selected based on a review of the literature and their theoretical justification. (Table 1)

### Data analysis

The analysis of the secondary data was conducted using Stata version 17 software. The sample data was summarized using descriptive statistics, specifically frequencies and percentages. The data for the 2020 GDHS were obtained through the utilization of a stratified multi-stage cluster sampling technique. This indicates that the data exhibit a hierarchical structure, wherein children are nested within clusters. The survey (‘svy’) module was used to adjust for stratification, clustering and sampling weights to compute the estimates of diarrhea among under-five children in The Gambia. In addition, the interdependence among individual children has significant implications for the analysis. Children who belong to the same cluster exhibit a higher degree of similarity among themselves compared to children who belong to different clusters. This phenomenon can result in the generation of artificially significant findings due to the presence of underestimated standard errors.

To account for the clustering effect of the sample, a multivariable multilevel logistic regression model was used. This type of model can account for a lack of independence across levels of nested data. The model was written as follows:

$$\text{l}\text{o}\text{g}\left[\frac{{\pi }_{ij}}{1-{\pi }_{ij}}\right]={\beta }_{0}+{\beta }_{1}{X}_{ij}+{\beta }_{2}{Y}_{ij}+{\beta }_{3}{Z}_{ij}+{u}_{j}+{e}_{ij}$$

Where $i$ and $j$ are the level 1 (child) and level 2 (mother) and level 3 (household) units, respectively. $X$, $Y$ and $Z$ refer to child-level, mother-level, and household-level variables, respectively. ${\pi }_{ij}$is the probability of a child developing diarrhea in the ${t}^{t/t}$household. The $\beta$ are the fixed coefficients. ${\beta }_{0}$ is the intercept, or the effect on the probability of developing diarrhea in the absence of predictors. ${u}_{j}$ is the random effect, or the effect of the household on childhood diarrhea for the ${j}^{\text{th }}$ household. ${e}_{ij}$ is the random error at the individual levels.

By assuming each household had different intercepts $\left({\beta }_{0}\right)$ and fixed coefficients $\left(\beta \right)$, the hierarchical (clustered) data nature and the within- and between-household variations were taken into account. Four sequential models were tested:

1. The null model, which did not include any explanatory variables (empty model) and only divided the total variance into child, mother, and household components.
2. The second model included only child-level variables.
3. The third model included only mother-level variables.
4. The fourth included only household-level variables.
5. Then the final model included all child-level, mother-level, and household-level variables.

The adjusted odds ratios (AORs) along with their corresponding 95% confidence intervals (CIs) were utilized to present the outcomes of the fixed effects. A *P*-value equal to or less than 0.05 was deemed to be statistically significant.

The statistical indicators of variability (random effects) encompassed variance, intracluster correlation Coefficient (ICC), a variance partition coefficient (VPC), Akaike information criteria (AIC), and Bayesian information criteria (BIC). The ICC was computed in order to assess whether the variability in childhood diarrhea is predominantly attributable to differences within households or between households. The ICC was calculated using the linear threshold method, as specified by Snijders and Bosker’s formula.: $C=\frac{{V}_{A}}{{V}_{A}+{\pi }^{2}/{/}_{3}}=\frac{{V}_{A}}{{V}_{A}+3.29}$, where ${V}_{A}$ is the estimated variance in each model [28]. VPC wass used to measure the total variation childhood diarrhea attributed to individual and cluster level factors in the multilevel model. It was computed using the formula: $VPC=\frac{{V}_{A}-{V}_{A}}{{V}_{A}}$, where ${V}_{A}=$ variance of the initial model, and ${V}_{B}=$ variance of the model with more terms.

The existence of multicollinearity was tested using the variance inflation factor (VIF). A VIF of > 5 is generally considered to be indicative of multicollinearity. However, in this study, the mean VIF was 1.28, the maximum VIF was 1.00, and the minimum VIF was 2.47. These values suggest that there is no evidence of multicollinearity between the independent variables.

### Variable selection and measurement

The variables for this study are presented in Table 1.

**Table 1:** Measurement of variables

Variables	Description
***Outcome Variable***	***Coding***
Diarrhea	0. No 1. Yes
***Independent Variable***	
Maternal age	2. < 20 years 3. 20–29 years 4. 30–30 years 5. 40–49 years
Residence	1. Urban 2. Rural
Mother’s education	0. No formal education 1. Primary 2. Secondary 3. Tertiary
Birth type	1 Single birth 2 Multiple births
Mother’s occupation	1. Employed 2. Not employed
Place of delivery	1. Home 2. Government hospital 3. Private hospital 4. Other places
Wealth Index	1. Poorest 2. Poorer 3. Middle 4. Richer 5. Richest
Religion	1. Christian 2. Islam 3. Traditionalist 4. Others
Health insurance coverage	0. No 1. Yes
Household has refrigerator	0. No 1. Yes
Sex of child	1. Male 2. Female
Toilet facilities	1. Unimproved 2. Improved
Family Structure	1. Monogamy 2. Polygamy
Water sources	1. Unimproved 2. Improved

### Ethical approval

The data in this manuscript was not collected by the authors themselves. Subsequently, authorization was acquired from the MEASURE DHS website to gain access to the data. Upon evaluation and approval in June 2022, the authors’ request for access to the data was granted. Ethics Committee, as well as the Ethics Boards of various partner organizations in different countries, including the Ministry of Health. The methodologies utilized in the surveys were implemented in adherence to relevant guidelines and regulations. During the course of the surveys, the female participants duly granted their informed consent.

## Results

At child-level variables, child sex, place of delivery/birth, preceeding birth interval and birth type were not significantly different in the prevalence of diarrhea as shown in Table 2. Child sex did not significantly affect the incidence of diarrhea (χ^2^ = 0.884, *p* = .347), with comparable rates among males (53.2%) and females (46.8%). Birthplace had a notable impact, albeit not statistically significant, with children born in government health centers displaying slightly higher diarrhea rates (76.6%) than those born at home (18.3%) or in private hospitals (3.4%; χ^2^ = 7.188, *p* = .066). The birth interval did not reveal a clear pattern, but children born after less than a two-year interval had slightly higher diarrhea percentages (14.2%) than those with longer intervals (χ^2^ = 7.619, *p* = .055). The type of birth (single or multiple) showed negligible differences in diarrhea rates (χ^2^ = 0.225, *p* = .635).

**Table 2:** Child-level variables

Variables	Frequency *N* (%)	Diarrhea (%)		*p*-value (χ^2^)
		No	Yes	
**Sex of Child**				
Male	3740 (51.9)	51.7	53.2	0.347 (0.884)
Female	3215 (48.1)	48.3	46.8	
**Place of delivery/birth**				
Home	1431 (20.1)	20.5	18.3	0.066 (7.188)
Government Health Centers	5346 (74.9)	74.6	76.6	
Private Hospitals	268 (3.8)	3.8	3.4	
Other Place	88 (1.2)	1.1	1.7	
**Preceeding birth interval**				
< 2	750 (12.7)	12.3	14.2	0.055 (7.619)
2	2440 (41.2)	41.9	38.0	
3	1382 (23.3)	23.0	24.9	
4+	1352 (22.8)	22.8	23.0	
**Birth type**				
Single birth	6998 (97.3)	97.2	97.5	0.635 (0.225)
Multiple births	197 (2.7)	2.8	2.5	

At mother-level variables, significant differences were found in the mother’s age at birth and incidence of diarrhea (χ2 = 22.937, *p* < .001). More specifically, mothers under 20 years of age had higher rates of children with diarrhea (12.7%) than those without diarrhea (9.9%). Other age groups showed varying incidences, but the pattern suggests a decrease in the occurrence of diarrhea with increasing maternal age. Other factors, such as religion, place of residence, and maternal education, were not statistically significant, as shown in Table 3.

**Table 3:** Mother-level variables

Variables	Frequency *N* (%)	Diarrhea (%)		*p*-value (χ^2^)
		No	Yes	
**Mother’s age at birth**				
< 20 years	751 (10.4)	9.9	12.7	< 0.001 (22.937)
20–29	3762 (52.3)	51.7	54.7	
30–39	2342 (32.6)	33.4	29.2	
40–49	340 (4.7)	5.0	3.4	
**Religion**				
Christianity	7126 (99.0)	99.0	99.1	0.874 (0.025)
Islam	69 (1.0)	1.0	0.9	
**Place of Residence**				
Urban	3182 (44.2)	44.6	42.6	0.177 (1.822)
Rural	4013 (55.8)	55.4	57.4	
**Education of mother**				
No education	3932 (54.7)	54.8	54.1	0.403 (2.925)
Primary	1336 (18.6)	18.4	19.2	
Secondary	1748 (24.3)	24.2	24.8	
Post-secondary	179 (2.4)	2.6	1.9	
**Maternal Occupation**				
Employed	3921 (54.5)	54.3	55.3	0.527 (0.400)
Unemployed	3274 (45.5)	45.7	44.7	

At household-level variables, a significant association was observed between household wealth quintile and diarrhea occurrence (*p*-value = 0.001, χ^2^=17.695), with the poor showing a higher incidence of diarrhea compared to other wealth classes. No significant association was found between diarrhea occurrence and water sources (*p*-value = 0.539, χ2 = 0.378), toilet facilities (*p*-value = 0.067, χ2 = 3.352), health insurance coverage (*p*-value = 0.661, χ2 = 0.1927), sex of the household head (*p*-value = 0.120, χ2 = 2.415), and family structure (*p*-value = 0.824, χ2 = 0.049) as shown in Table 4.

**Table 4:** Household-level variables

Variables	Frequency *N* (%)	Diarrhea (%)		*p*-value (χ^2^)
		No	Yes	
**Household Wealth Quintile**				
Poor	2427 (36.3)	35.2	41.0	0.001 (17.695)
Poorer	1433 (21.4)	21.9	19.5	
Middle	1283 (19.2)	19.7	17.2	
Richer	889 (13.3)	13.2	12.6	
Richest	653 (9.8)	10.0	8.7	
**Water sources**				
Unimproved	491 (6.8)	6.9	6.4	0.539 (0.378)
Improved	6704 (93.2)	93.1	93.6	
**Toilet facility**				
Unimproved	3213 (44.7)	45.2	42.5	0.067 (3.352)
Improved	3982 (55.3)	54.8	57.5	
**Health Insurance coverage**				
No	7073 (98.3)	98.3	98.4	0.661 (0.1927)
Yes	22 (1.7)	1.7	1.6	
**Sex of Household head**				
Male	6290 (87.4)	87.1	88.7	0.120 (2.415)
Female	905 (12.6)	12.9	11.3	
**Family structure**				
Monogamy	4509 (62.7)	62.7	62.4	0.824 (0.049)
Polygamy	2686 (37.3)	37.3	37.6	

### Multi-level fixed effects of contextual factors associated with childhood diarrhea

The analysis of factors at the child-level variables that relate to childhood diarrhea shows significant differences across regions and places of delivery as shown in Table 5. When compared to Banjul, which is used as a reference region, children in Kerewan exhibit statistically lower odds of experiencing childhood diarrhea (aOR = 0.61; 95% CI = 0.38–0.98). In relation to places of delivery, children born in government health centers, as compared to those born at home, have a significantly higher likelihood of diarrhea (aOR = 1.29; 95% CI = 1.05–1.58). The effects of different mother-level variables shows that regions such as Kanifing (aOR = 0.63 (95%; CI = 0.41–0.97), Kerewan (aOR = 0.49; 95% CI 0.30–0.79), Janjanbureh (aOR = 0.60; 95% CI = 0.37–0.97), and Basse (aOR = 0.53; 95% CI 0.33–0.83) has reduce odds of childhood diarrhea compared to those from Banjul. Mothers with post-secondary education compared to those with no education were associated with a significantly lower odds of childhood diarrhea, (aOR = 0.58; 95% CI 0.35–0.94). Among household-level variables, several regions such as Kanifing (aOR = 0.64, 95% CI 0.41–0.98), Kerewan (aOR = 0.50, 95% CI 0.32–0.58), Janjanbureh (aOR = 0.62, 95% CI 0.39–0.99), and Basse (aOR = 0.55, 95% CI 0.35–0.86) showed statistically lower odds of childhood diarrhea than Banjul, indicating a decreased risk in these regions.

In the final (full) model, Kerewan (aOR = 0.58; 95% CI = 0.33–0.98) and Basse (aOR = 0.59; 95% CI = 0.35–0.98) had significantly lower odds of childhood diarrhea compared to Banjul, signifying reduced risk in these areas. Regarding the child’s sex, female children show slightly lower, yet significant, odds of diarrhea compared to males (aOR = 0.96; 95% CI = 0.86–0.98). For birthplace, deliveries at government health centers are associated with higher odds of childhood diarrhea compared to home births (aOR = 1.24; 95% CI = 1.01–1.52). Mothers with post-secondary education have significantly lower odds of having children with diarrhea, as compared to those without any education (aOR = 0.50; 95% CI = 0.26–0.99).

### Random effects for measures of associations

The analysis began with an empty model (Model 0), which exhibited a variance of 0.34 (SE = 0.06, significant at *p* < .05). The random effects variance decreased as child-level variables (Model 1), mother-level variables (Model 2), and household-level variables (Model 3) were sequentially added to the models. This drop in variance indicated that these variables contributed to the occurrence of childhood diarrhea. However, the variance remained stable from Model 2 to Model 4 (full model) at 0.24, showing that the addition of household-level variables did not significantly change the model’s explanatory power. The VPC increased from 4.9% in the empty model to 6.6% in the full model, indicating a slight increase in the proportion of total variance attributed to the cluster level. The ICC increased from 0.171 in the empty model to 0.177 in the full model, suggesting a minor increase in the proportion of total variance due to between-cluster differences. In terms of model fit, AIC and BIC both decreased from the empty model to the full model, suggesting that the full model with all variables included provided the best fit to the data. In particular, the full model had an AIC of 5041.9 and a BIC of 5312.1, which were lower than those of any other model. In conclusion, the full model incorporating child, mother, and household-level variables provided the best explanation for the occurrence of childhood diarrhea in The Gambia, highlighting the multifactorial nature of this health issue.

**Table 5:** Contextual factors associated with childhood diarrhea

Fixed effects	Model 0	Model 1	Model 2	Model 3	Model 4
	Empty model (OR, CI)	Child-level variables (aOR, CI)	Mother-level variables (aOR, CI)	Household-level variables (aOR, CI)	Full model (aOR)
**Region**					
Banjul		1.0 (RC)	1.0 (RC)	1.0 (RC)	1.0 (RC)
Kanifing		0.63 (0.38, 1.05)	0.63* (0.41, 0.97)	0.64* (0.41, 0.98)	0.66 (0.40, 1.09)
Brikama		0.93 (0.60, 1.44)	0.85 (0.57, 1.26)	0.82 (0.55, 1.21)	0.96 (0.62, 1.50)
Mansakonko		0.98 (0.60, 1.60)	0.78 (0.47, 1.29)	0.83 (0.52, 1.33)	0.87 (0.50, 1.53)
Kerewan		0.61* (0.38, 0.98)	0.49 *(0.30, 0.79)	0.50* (0.32, 0.58)	0.58* (0.33, 0.98)
Kuntaur		1.52 (0.95, 2.42)	1.14 (0.69, 1.88)	1.15 (0.73, 1.83)	1.31 (0.75, 2.29)
Janjanbureh		0.73 (0.45, 1.17)	0.60* (0.37, 0.97)	0.62* (0.39, 0.99)	0.68 (0.39, 1.1)
Basse		0.63 (0.39, 1.00)	0.53* (0.33, 0.83)	0.55* (0.35, 0.86)	0.59* (0.35, 0.98)
**Sex of Child**					
Male		1.0 (RC)			1.0 (RC)
Female		0.96 (0.82, 1.12)			0.96* (0.86, 0.98)
**Place of delivery**					
Home		1.0 (RC)			1.0 (RC)
Government Health Centers		1.29* (1.05, 1.58)			1.24* (1.01, 1.52)
Private Hospitals		1.06 (0.63, 1.77)			1.01 (0.58, 1.71)
Other Place		1.68 (0.89, 3.19)			1.57 (0.82, 2.98)
**Preceeding birth interval**					
< 2		1.0 (RC)			1.0 (RC)
2		0.79 (0.62, 1.00)			0.82 (0.64, 1.05)
3		0.96 (0.74, 1.24)			1.01 (0.77, 1.31)
4+		0.92 (0.71, 1.20)			0.95 (0.72, 1.25)
**Birth type**					
Single birth		1.0 (RC)			1.0 (RC)
Multiple births		0.67 (0.41, 1.08)			0.84 (0.51, 1.37)
**Mother’s age at birth**					
< 20 years			1.0 (RC)		1.0 (RC)
20–29			1.19 (0.93, 1.53)		0.84 (0.55, 1.30)
30–39			1.33 (0.98, 1.80)		0.91 (0.57, 1.47)
40–49			1.16 (0.73, 1.83)		0.76 (0.42, 1.38)
**Religion**					
Islam			1.0 (RC)		1.0 (RC)
Christianity			1.02 (0.49, 2.12)		1.09 (0.49, 2.42)
**Place of Residence**					
Urban			1.0 (RC)		1.0 (RC)
Rural			1.22 (0.49, 2.12)		1.09 (0.77. 1.54)
**Education of mother**					
No education			1.0 (RC)		1.0 (RC)
Primary			1.01 (0.83, 1.21)		1.01 (0.80, 1.24)
Secondary			0.94 (0.78, 1.13)		0.97 (0.78, 1.21)
Post-secondary			0.58* (0.35, 0.94)		0.50* (0.26, 0.99)
**Maternal Occupation**					
Employed			1.0 (RC)		1.0 (RC)
Unemployed			0.89 (0.77, 1.04)		0.94 (0.79, 1.11)
**Water sources**					
Unimproved				1.0 (RC)	1.0 (RC)
Improved				1.01 (0.73, 1.37)	0.99 (0.70, 1.41)
**Toilet facility**					
Unimproved				1.0 (RC)	1.0 (RC)
Improved				1.13 (0.95, 1.35)	1.12 (0.92, 1.37)
**Household Wealth Quintile**					
Poor				1.0 (RC)	1.0 (RC)
Poorer				0.88 (0.70, 1.09)	0.85 (0.66, 1.10)
Middle				0.84 (0.65, 1.08)	0.77 (0.56. 1.05)
Richer				1.01 (0.74, 1.35)	0.87 (0.60, 1.28)
Richest				0.74 (0.52, 1.06)	0.73 (0.46, 1.16)
**Health Insurance coverage**					
No				1.0 (RC)	1.0 (RC)
Yes				0.97 (0.56, 1.69)	1.16 (0.59, 2.27)
**Sex of Household head**					
Male				1.0 (RC)	1.0 (RC)
Female				0.90 (0.72, 1.12)	0.84 (0.64, 1.10)
**Family structure**					
Monogamy				1.0 (RC)	1.0 (RC)
Polygamy				1.03 (0.89, 1.19)	1.16 (0.98, 1.38)
***Random Effects***	**Empty**	**Child**	**Mother**	**Household**	**Final**
Variance (SE)	0.34 (0.06) *	0.25 (0.06) *	0.24 (0.05) *	0.24 (0.05)	0.24 (0.06) *
VPC (%)	4.9	6.4	5.8	5.9	6.6
Log likelihood	-3184.4	-2514.3	-3127.1	-3165.3	-2480.4
***Model fit statistics***					
ICC	0.171	0.180	0.154	0.152	0.177
AIC	6374.7	5066.8	6300.2	6368.6	5041.9
BIC	6395.1	5191.9	6456.7	6497.9	5312.1

*P < .05, AIC = Akaike Information Criterion, BIC = Bayesian Information Criteria, OR = Odds Ratio, AOR = Adjusted Odds Ratio, SE = Standard Error, VPC = Variance Partition Coefficient

## Discussion

The occurrence of diarrhea at the child level was found to be significantly associated with the region and place of birth. The educational attainment of mothers and the geographical region in which they reside were found to be significant factors at the individual level, specifically in relation to the mothers themselves. However, at the household level, only the geographical region was found to have a significant impact. The findings of this study revealed that the intra-class correlation (ICC) values exceeded 10% for all models pertaining to childhood diarrhea. This observation underscores the necessity of employing a multilevel study design [21].

The likelihood of diarrhea in female children was found to be less than that in male children. This result is consistent with a cross-sectional study conducted in Ethiopia, which found that boys had 2.52 times greater odds of experiencing acute diarrhea than girls [22]. Similar findings have been reported in recent studies from SSA including Nigeria [23, 24]. The reason for this gender disparity in the incidence of diarrhea illness remains unclear, despite numerous studies highlighting its prevalence in many developing countries [25]. The disparity is hypothesized to stem from factors tied to gender, such as biological differences, environmental exposure, and cultural influences. One environmental hypothesis proposes that boys, particularly older ones, might have different exposure levels due to activities such as spending more time away from home or accompanying their fathers to work, which may expose them to infectious pathogens more frequently [22, 26]. On the biological front, the hypothesis suggests that there may be physiological differences between girls and boys in relation to acute diarrhea, making boys more susceptible [27, 28].

The educational attainment of a mother appears to significantly influence the likelihood of childhood diarrhea. The results of this study show that children of mothers who have received post-secondary education are less likely to have diarrhea than children of mothers without education. This aligns with prior research indicating that children of less-educated mothers in East Africa [29], Ethiopia [30], Nigeria [31], and Ghana [32] had higher odds of experiencing childhood diarrhea. This could be attributed to the fact that mothers with more education are likely to have better knowledge, attitudes, and access to the crucial health information needed to effectively prevent diarrhea. Another likely explanation is that education is anticipated to enhance healthcare and hygiene practices within households. It can equip parents with the necessary knowledge about the prevention and transmission of diarrhea.

Mothers residing in the Kerewan and Basse regions demonstrated lower instances of diarrhea in children under five years, which could be attributed to cultural differences and health-seeking behaviors, compared to mothers from Banjul. These findings are similar to those of a previous study in Gambia, where rural regions reported reduced odds of under-five diarrhea cases compared to urban areas, such as Banjul [33]. Although the underlying factors specific to these regions have not been extensively studied, the authors suggest that this finding warrants further investigation. Possible explanations for these regional variations could include disparities in sociocultural norms, healthcare accessibility and quality, community participation, stakeholder commitment, and implementation of maternal and child healthcare services.

In this study, place of delivery was significantly associated with under-five diarrhea, as those born at government health centers had increased odds of diarrhea cases compared to those born at home. This implies that children born at government health centers have a higher probability of experiencing diarrhea than those born at home. This information is essential for identifying high-risk groups and creating targeted interventions to reduce childhood diarrhea. It also underlines the importance of focusing not only on individual-level factors but also on regional and health infrastructure-related factors when studying and addressing childhood diarrhea. Further investigations are needed to understand the underlying mechanisms of these associations and develop targeted interventions. There are several possible reasons why diarrhea might be more likely among children delivered in hospitals compared to those delivered at home. Firstly, could be due to hospital-acquired infections. Hospitals are environments where infectious agents are more prevalent, increasing the risk of exposure to pathogens that can cause diarrhea. In hospitals, there is a higher likelihood of antibiotic use, which can disrupt the natural balance of gut bacteria and lead to diarrhea. Hospitals may not always have the same level of sanitation as homes, increasing the risk of exposure to contaminated water or food, which can cause diarrhea. Children who are hospitalized may already have underlying health conditions that make them more susceptible to diarrhea. Moreover, it is important to note that these are potential factors, and the actual reasons can vary depending on the specific hospital practices and circumstances.

### Strength and limitation

One advantage of this study is that it utilizes a large, representative dataset from Gambia, which lends credibility to the findings and allows them to be generalized across the country. Another strength of this research is the application of multilevel modeling, a statistical model that accommodates the hierarchical structure of the data and provides more reliable estimates. However, this study had certain limitations. First, the data on childhood diarrhea were self-reported, introducing the potential for recall bias, even though the recall period for illnesses was limited to two weeks prior to the survey. Second, the analyses were based on cross-sectional survey data from the GDHS, which did not allow causal inferences. Third, this study could not capture the impact of seasonal changes on diarrhea morbidity because of the nature of the cross-sectional GDHS data used. Fourth, data from different time frames were combined, assuming minimal changes in demographic characteristics over a 5-year period. Finally, the results could have been influenced by confounders that weren’t measured due to the derivative nature of the data. However, despite these limitations, the utilization of a multilevel model provided an account for the clustered nature of the GDHS data, thereby enhancing the precision of the estimates. Furthermore, the application of nationally representative GDHS data can amplify the generalizability of the results.

## Conclusion

The research underscores the fact that factors influencing childhood diarrhea are multi-layered, encompassing not just the geographical region, but also characteristics related to both the child and mother. On the child’s side, aspects like birthplace and gender play a role, while on the mother’s side, education level is statistically significant in relation to childhood diarrhea in Gambia. The study suggests that it’s essential to take into account these changeable factors when enhancing existing health interventions to improve child health outcomes in the country.

### Electronic supplementary material

Below is the link to the electronic supplementary material.

Supplementary Material 1

## Data Availability

Data for this study were sourced and available here: http://dhsprogram.com/data/available-datasets.cfm.
